# Multiple major QTL lead to stable yield performance of rice cultivars across varying drought intensities

**DOI:** 10.1186/1471-2156-15-16

**Published:** 2014-02-03

**Authors:** Shalabh Dixit, Anshuman Singh, Ma Teresa Sta Cruz, Paul T Maturan, Modesto Amante, Arvind Kumar

**Affiliations:** 1 International Rice Research Institute, DAPO Box 7777, Metro Manila, Philippines

**Keywords:** Rice, Drought, Grain yield, QTL, Stability

## Abstract

**Background:**

Availability of irrigation water is becoming a major limiting factor in rice cultivation. Production in rainfed areas is affected in particular by drought events, as these areas are commonly planted to high-yielding drought-susceptible rice (*Oryza sativa* L.) varieties. The use of bulk segregant analysis (BSA), taking grain yield (GY) as a selection criterion, has resulted in the identification of several large-effect QTL. A QTL mapping study was undertaken on a BC_1_F_3:4_ population developed from the cross IR55419-04/2*TDK1 with the aim of identifying large-effect QTL in the background of TDK1, a popular variety from Lao PDR.

**Results:**

The study identified three QTL—*qDTY*_*3.1*_ (RM168-RM468), *qDTY*_*6.1*_ (RM586-RM217), and *qDTY*_*6.2*_ (RM121-RM541)—for grain yield under drought. *qDTY*_*3.1*_ and *qDTY*_*6.1*_, showed consistent effect across seasons under lowland drought-stress conditions while *qDTY*_*6.1*_ and *qDTY*_*6.2*_ showed effect under both upland and lowland drought conditions. The test of QTL effect, conducted through a QTL class analysis, showed the complimentary nature of *qDTY*_*3.1*_ and *qDTY*_*6.1*_. Both QTL showed specific patterns of effect across different maturity groups within the mapping population and higher stability for grain yield was seen across stress levels for lines with both QTLs as compared to those with single or no QTL.

**Conclusions:**

The study offers a clear understanding of large-effect QTL for grain yield under drought and their effect as individual QTL and in various combinations. The study also opens up an opportunity to develop a drought-tolerant version of TDK1 through marker-assisted backcross breeding and has led to a large-scale QTL pyramiding program aiming to combine these QTL with *Sub1* in the background of TDK1 as recipient variety.

## Background

Availability of irrigation water is becoming the major factor that limits rice cultivation [1] in both rainfed and irrigated ecosystems. In rainfed ecosystems, the problem of water shortage due to early withdrawal or failure of monsoon rains or due to a long period between two rains has persisted for centuries. In recent years, drought incidence and severity has increased because of climate change-related processes [2,3]. Growing demand for water from an expanding industrial sector, in addition to increasing residential requirements, has rapidly reduced available water for agriculture [4], thereby increasing the intensity of drought in rainfed ecosystems and aggravating water shortage in irrigated ecosystems. This scenario demands the development and dissemination of ecosystem-specific water-saving technologies that can reduce water requirements of the rice crop without a yield penalty. These technologies can include new varieties that can produce good yield and entail only minimal yield loss under water-deficient conditions. Ironically, a large part of rainfed ecosystems is still planted to varieties that were developed particularly for irrigated lowland ecosystems [5]. These varieties require a continuous supply of water throughout the season and risk heavy yield loss if drought occurs [6]. Even in the irrigated ecosystem, cultivation of irrigated varieties that are suitable to puddled- transplanted conditions under water-saving techniques (such as dry direct seeding) results in a yield penalty. Recent research has shown that varieties developed through direct selection for grain yield (GY) under drought-stress and non-stress conditions from progenies derived from crosses of drought-tolerant donors and high-yielding drought-susceptible varieties provide a yield advantage under drought [6-8], in addition to maintaining a high yield potential under non-stress conditions. These findings led to a series of QTL-identification studies on a wide range of donor and recipient parents, resulting in the identification of several large-effect QTL for grain yield under varying severities of reproductive-stage drought-stress for both direct-seeded upland and transplanted lowland ecosystems [5,9-14]. With the availability of these QTL, several marker-assisted breeding (MAB) programs have begun work for pyramiding the QTL into high-yielding popular varieties. The studies indicate the importance of the QTL regions not only in improving current varieties for yield under drought following MAB but also in developing new drought-tolerant varieties following marker-assisted selection (MAS), and in deciphering the physiological and molecular mechanisms behind the yield advantage conferred by these QTL.

While the identified QTL are a valuable source of drought tolerance, it is important for the success of MAS that the effect of these QTL under varying drought intensities be understood clearly. In the past, it had been found that the effect of major GY QTL differ across varying drought intensities. For example, Bernier et al. [15] reported an increasing effect of *qDTY*_*12.1*_ on GY with increasing intensity of drought. Similarly, Swamy et al. [16] reported the effect of specific combinations of QTL on GY under drought. In this study, the GY of lines with two drought QTL was found to be higher than that of lines with three or four QTL. It also becomes important to understand the effect of these QTL on other traits, such as days-to-50%-flowering (DTF) and plant height (PH), to find any possible effect of the QTL on the plant type and phenology of the developed lines. These traits play an important role in deciding the target ecosystems for developed lines, as suitability of lines to a particular ecosystem depend on duration and plant type. Short-duration lines, for example, are more suitable to rainfed upland areas while medium- to late-duration lines are suitable for rainfed lowlands.

Our study was undertaken to identify large-effect QTL for grain yield under drought in the background of TDK 1, a popular lowland variety in Lao PDR. This study also reports the standardization of effect of specific QTL combinations that lead to maximum stability of lines across varying stress intensities and under non-stress conditions. Finally, an attempt was made to understand the effect of the identified QTL on DTF to determine if it leads to any desirable or undesirable effect on GY under stress and non-stress conditions.

## Results

### Phenotypic variation in upland and lowland ecosystems

Table 1 summarizes the results of statistical analysis conducted on the IR55419-04/2*TDK1 population. Six experiments (five under lowland and one under upland conditions) were conducted using this population. The three stress experiments conducted under lowland conditions had the following mean GY: 1556 kg ha^-1^ from the lowland severe-stress (LSS) experiment (DS2011), and 2547 kg ha^-1^ and 2116 kg ha^-1^ from the lowland moderate stress (LMS) I (DS2012) and LMS II (DS2013), respectively. The three experiments showed yield reductions (YR) of 70%, 51%, and 57%, respectively, compared with the lowland non-stress (LNS) I and II experiments that had a mean GY of 5237 kg ha^-1^ (DS2012) and 4965 kg ha^-1^ (DS2013) respectively. The upland mild-stress (UMiS) experiment (WS2012) had a mean GY of 3528 kg ha^-1^. The yield reduction from this experiment (33%) and LSS experiment was derived from comparison with the LNS experiment conducted in DS2012, due to the absence of non-stress counterparts of these two experiments. Moderate-to-high heritability was seen in GY for this population (Table 1).

**Table 1:** Details of experiments conducted under upland and lowland drought stress and non-stress conditions for the identification of QTL

**Population size**	**Season**	**Environment**	**GY (kg ha**^**-1**^**)**			**DTF**			**PH (cm)**			**YR**
			**M**	**H**	**P**^**a**^	**M**	**H**	**P**	**M**	**H**	**P**	
365	DS2011	LSS	1556 ± 357	0.80	****	86 ± 2	0.88	****	97 ± 5	0.82	****	70^b^
365	DS2012	LMS I	2547 ± 537	0.46	****	79 ± 3	0.86	****	91 ± 6	0.65	****	51
365	DS2013	LMS II	2116 ± 466	0.65	****	83 ± 4	0.56	****	92 ± 8	0.37	****	57
365	DS2012	LNS I	5237 ± 789	0.67	****	74 ± 4	0.14	*	119 ± 7	0.74	****	
365	DS2013	LNS II	4965 ± 629	0.78	****	74 ± 2	0.87	****	109 ± 7	0.71	****	
100	WS2012	UMiS	3528 ± 673	0.93	****	84 ± 2	0.98	****	107 ± 5	0.81	****	33^b^

LSS: lowland severe stress; LMS: lowland moderate stress; LNS: lowland non-stress, UMiS: upland mild stress.

means ± SED (M), broad-sense heritability (H), P values (P), and percentage yield reduction (YR) for grain yield (GY, in kg ha^-1^), days to 50% flowering (DTF), and plant height (PH, in cm).

^a^: probability of difference between genotypes; *, ****: significant at 5%, and 0.01% *P* levels, respectively; ^b^: compared with the DS2012 LNS trial.

The mean DTF for the population was recorded as 86, 79, 83, 74, 74, and 84 from the LSS, LMS I, LMS II, LNS I, LNS II, and UMiS experiments, respectively. High *H* estimates and significance (at the 0.01% *P* level) were seen for DTF in a majority of the experiments. However, a low *H* estimate (0.14) and significance (at the 0.05% *P* level) were seen for DTF in the LNS I experiment (Table 1). Heavy rains throughout the flowering period in this experiment severely affected the crop, and could be one of the reasons for the low heritability and significance of DTF. A general trend of high variation and heritability in the population, can however be seen across drought-stress and non-stress conditions from the other five experiments (Table 1).

The mean PH for the population was recorded as 97, 91, 92, 119, 109, and 107 cm from the LSS, LMS I, LMS II, LNS I, LNS II, and UMiS experiments, respectively. All experiments showed a high significance for PH (at the 0.01% *P* level) and *H* estimates (Table 1).

### Bulk segregant analysis (BSA) and QTL mapping

**Figure 1:**
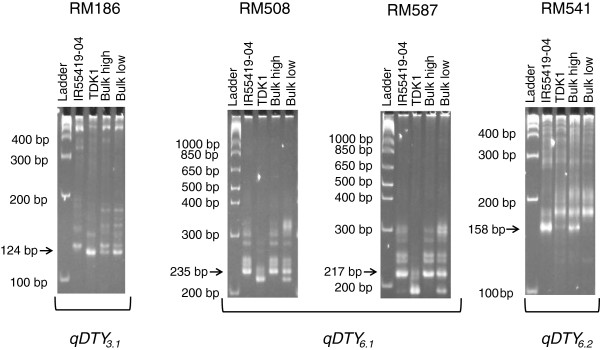
**BSA results for RM186 (*qDTY***_***3.1 ***_**region), RM587 and RM508 (*qDTY***_***6.1 ***_**region) and RM541 (*qDTY***_***6.2 ***_**region) for high- and low-yielding bulks identified from screening under LSS conditions in DS2011.** BSA: bulk segregant analysis, LSS: lowland severe stress.

Bulk segregant analysis of the IR55419-04/2*TDK1 population revealed four markers—RM186 on chromosome 3 and RM587, RM508, and RM541 on chromosome 6 indicating clear polymorphism between the bulks and the parents. A clear pattern of similarity was found between the high-yielding bulks with IR55419-04 and the low-yielding bulks with TDK1 bands (Figure 1). These results indicate the possible linkage of these markers to QTL affecting GY under lowland drought. The banding patterns of these markers also indicate that the positive allele at all four marker loci is contributed by IR55419-04. The GY data of the lines used to develop the high- and low-yielding bulks in the DS2011 and subsequent seasons are presented in Additional file 1: Table S1. Similar to DS2011, the two sets of lines showed very contrasting results in the experiments, indicating a significant effect from one or more of the identified QTL.

**Figure 2:**
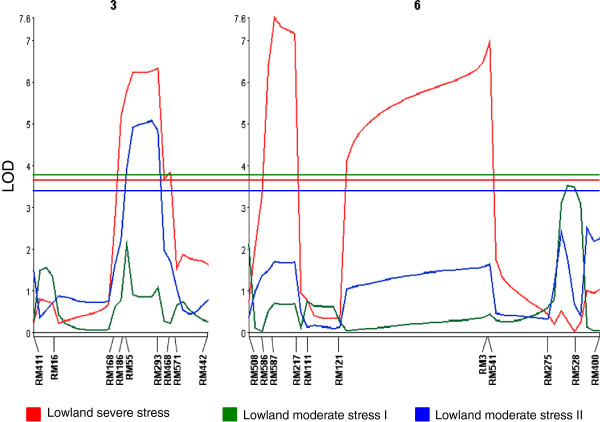
**QTL likelihood curves of LOD scores for grain yield (GY) under lowland drought stress conditions for chromosomes 3 and 6.** Lowland severe-stress (LSS), Lowland moderate-stress (LMS). Marker loci at their respective cM positions are on the X axis and LOD scores are on the Y axis. Horizontal lines correspond to the LOD threshold for the respective experiments (color-coded).

Composite interval mapping (CIM) analysis conducted on the population showed the presence of three QTL—*qDTY*_*3.1*_*, qDTY*_*6.1*_*,* and *qDTY*_*6.2*_*—*for GY under drought (Table 2, Figure 2)*.* All three QTL showed an effect on GY under LSS conditions, with *qDTY*_*3.1*_ explaining 7.9% of the phenotypic variance and showing a LOD score of 6.3 and an additive effect of 165.3 kg. The QTL was identified at 124 cM, flanked by RM168 and RM468, with RM293 as the marker closest to the LOD peak. *qDTY*_*6.1*_ was identified at 9.7 cM with RM586 and RM217 as the flanking markers and RM587 as the marker closest to the LOD peak. The QTL explained 9.3% of the phenotypic variance, had a LOD score of 7.5 and an additive effect of 188.6 kg. The third QTL, *qDTY*_*6.2*_, was identified at 75.7 cM, flanked by RM121 and RM541, with RM3 as the marker closest to the LOD peak. The QTL explained 8.6% of the phenotypic variance and had a LOD score of 7.0 and an additive effect of 260.8 kg.

**Table 2:** QTL identified for grain yield (GY), days to 50% flowering (DTF), and plant height (PH) under upland and lowland conditions through CIM

**Trait**	**Ecosystem**	**Chromosome**	**Locus name**	**Interval**	**Peak marker**	**Peak position**	**LOD**	** A**	**R**^**2**^
GY	LSS	3	*qDTY*_*3.1*_	RM168-RM468	RM293	124.0	6.3	165.3	7.9
	LSS	6	*qDTY*_*6.1*_	RM586-RM217	RM587	9.7	7.5	188.6	9.3
	LSS	6	*qDTY*_*6.2*_	RM121-RM541	RM3	75.7	7.0	260.8	8.6
	LMS II	3	*qDTY*_*3.1*_	RM168-RM468	RM293	122.0	5.1	171.5	6.9
	LNS II	3	*qDTY*_*3.1*_	RM168-RM468	RM55	114.0	9.0	−377.4	10.7
	LNS II	6	*qDTY*_*6.3*_	RM528-RM400	RM528	105.7	4.3	307.6	5.2
	UMiS	3	*qDTY*_*3.1*_	RM168-RM468	RM293	124.0	3.5	627.0	15.0
	UMiS	6	*qDTY*_*6.1*_	RM586-RM217	RM587	9.7	9.5	976.4	35.6
	UMiS	6	*qDTY*_*6.2*_	RM121-RM541	RM3	75.7	4.8	1083.3	19.9
DTF	LSS	3	*qDTF*_*3.1*_	RM168-RM468	RM55	114.0	9.5	−1.7	11.3
	LSS	6	*qDTF*_*6.1*_	RM586-RM217	RM217	13.7	6.4	−1.4	7.7
	LMS I	3	*qDTF*_*3.1*_	RM168-RM468	RM55	114.0	10.0	−1.9	11.9
	LMS II	3	*qDTF*_*3.1*_	RM168-RM468	RM55	114.0	4.7	−1.1	5.8
	LMS II	6	*qDTF*_*6.1*_	RM586-RM217	RM217	15.7	3.7	−0.9	4.6
	LNS II	3	*qDTF*_*3.1*_	RM168-RM468	RM55	114.0	9.0	−1.3	10.8
	LNS II	6	*qDTF*_*6.1*_	RM586-RM217	RM217	13.7	5.7	−1.0	6.9
	UMiS	3	*qDTF*_*3.1*_	RM168-RM468	RM293	124.0	3.4	−3.4	14.6
	UMiS	6	*qDTF*_*6.1*_	RM586-RM217	RM587	9.7	6.7	−4.6	26.5
PH	LSS	6	*qDTH*_*6.1*_	RM275-RM528	RM528	103.7	6.4	3.3	7.8
	LMS I	3	*qDTH*_*3.1*_	RM293-RM571	RM468	128.0	3.4	−1.7	4.2
	LMS II	6	*qDTH*_*6.1*_	RM275-RM528	RM528	103.7	4.5	2.7	5.5
	LNS I	6	*qDTH*_*6.1*_	RM275-RM528	RM528	103.7	5.6	4.1	6.8
	LNSII	6	*qDTH*_*6.1*_	RM275-RM528	RM528	103.7	5.9	4.0	7.1

R^2^: percentage of phenotypic variance explained by marker closest to the peak; A: additive effect as percentage of trial mean; LSS: lowland severe stress; LMS: lowland moderate stress; LNS: lowland non-stress; UMiS: upland mild stress.

*qDTY*_*3.1*_ also showed an effect under LMS II. It was identified at 122 cM, flanked by RM168 and RM468, with RM293 as the marker closest to the LOD peak. The QTL explained 6.9% of the phenotypic variance and had a LOD score of 5.1 and an additive effect of 171.5 kg. All three QTL identified under LSS conditions (*qDTY*_*3.1*_, *qDTY*_*6.1,*_ and *qDTY*_*6.2*_*)* also showed a significant effect for GY under UMiS conditions (Table 2). In this experiment, *qDTY*_*3.1*_ explained 15.0% of the phenotypic variance and had a LOD score of 3.5 and an additive effect of 627.0 kg. *qDTY*_*6.1*_ explained 35.6% of phenotypic variance and had a LOD score of 9.5 and an additive effect of 976.4 kg. *qDTY*_*6.2*_ explained 19.9% of phenotypic variance and had a LOD score of 4.8 and an additive effect of 1083.3 kg. The yield-enhancing allele in all of these experiments was contributed by the tolerant parent IR55419-04. Surprisingly, *qDTY*_*3.1*_ also showed an effect in the LNS II experiment but the yield-enhancing allele was contributed in this case by the susceptible parent TDK1. The QTL explained 10.7% of phenotypic variance and had a LOD score of 9.0 and an additive effect of −377.4 kg. Another region, *qDTY*_*6.3*_, was also identified in this experiment at 105.7 cM, flanked by RM528 and RM400. The QTL explained 5.2% of phenotypic variance and had an LOD score of 4.3 and an additive effect of 307.6 kg. Several other QTL for DTF and PH were also identified from these two chromosomes (Table 2).

### Stability analysis

**Figure 3:**
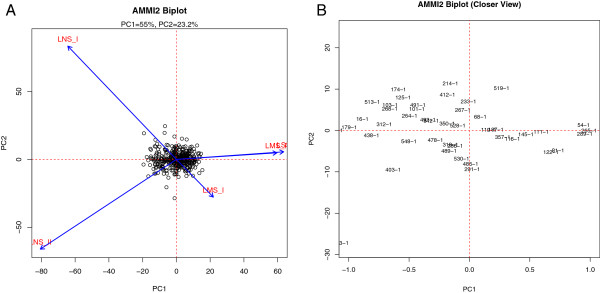
**AMMI biplot of grain yield showing the stability of lines across lowland stress and non-stress conditions. (A)** Full plot view **(B)** Magnified view showing the most stable lines. LSS: lowland severe stress; LMS: lowland moderate stress; LNS: lowland non-stress.

The GXE interaction of the mapping population was estimated across varying severity of stress during the three seasons. Analysis showed a highly significant GXE interaction effect with a p value <0.0001. Because of the high significance of GXE interactions of the mapping population, an AMMI analysis was conducted to identify the most stable lines (Figure 3). Table 3 presents the list of lines with the most stable performance across all seasons, based on the values of principal components (PC1 and PC2). The table also presents the mean GY of these lines in different environments, as well as the overall mean across environments. In order to understand the role of the two most consistent QTL (*qDTY*_*3.1*_ and *qDTY*_*6.1*_), the data was combined with information about the alleles of peak markers of the two QTL in these lines (Table 3). Out of the 18 most stable lines, 16 carried the donor allele of at least one of the two QTL at the peak marker position while the remaining two lines showed a heterozygote allele at the loci. Eight out of the 18 lines carried donor alleles at both loci. Even within this set of 18 lines, 5 out of the 6 most stable lines showed the presence of donor alleles at both loci.

**Table 3:** Grain yield (GY), days to 50% flowering (DTF), plant height (PH), and QTL content of the most stable lines across varying levels of lowland stress and non-stress conditions

**Line**	***qDTY***_***3.1***_	***qDTY***_***6.1***_	**DTF**		**PH**		**GY**					**Mean**	**PC1**	**PC2**
			**LSS**	**LNS**	**LSS**	**LNS**	**LSS**	**LMS I**	**LMS II**	**LNS I**	**LNS II**			
IR90266-B-54-1	H	-	88	72	87	107	1052	2250	2230	4877	4615	3005	0.9	−0.1
IR90266-B-116-1	-	+	82	74	99	111	1467	2391	1711	4664	4905	3028	0.4	−3.5
IR90266-B-264-1	H	+	89	76	88	109	1183	2272	1932	5191	4588	3033	−0.5	2.1
IR90266-B-268-1	+	-	85	73	89	105	1564	1576	2024	5290	4727	3036	−0.7	3.8
IR90266-B-438-1	H	H	81	75	96	114	1735	1882	1745	4802	5163	3065	−0.8	−2.6
IR90266-B-265-1	-	+	88	73	97	111	1423	2247	2324	4909	4940	3169	1.0	−1.4
IR90266-B-119-1	+	+	86	74	81	101	1077	2307	2546	4992	4975	3179	0.2	−1.2
IR90266-B-267-1	H	+	85	73	94	105	1491	1660	2692	5414	4946	3241	−0.1	3.5
IR90266-B-16-1	+	+	83	71	104	115	1618	2411	1969	5373	4942	3263	−0.9	1.3
IR90266-B-350-1	H	H	86	76	91	101	1421	2513	2351	5289	5000	3315	−0.2	0.3
IR90266-B-492-1	+	+	83	74	100	119	2604	2160	1485	5392	5153	3359	−0.3	1.2
IR90266-B-145-1	+	H	86	73	110	124	1285	2952	2574	5156	5132	3420	0.5	−2.3
IR90266-B-312-1	+	+	85	75	90	101	1733	3068	1873	5470	5020	3433	−0.7	0.1
IR90266-B-512-1	+	+	85	74	102	119	1790	2877	2132	5542	5057	3480	−0.3	1.0
IR90266-B-111-1	+	+	87	74	95	112	2572	2260	2254	5297	5580	3592	0.6	−1.8
IR90266-B-101-1	+	+	86	72	101	118	1539	2770	2774	5892	5053	3606	−0.4	3.8
IR90266-B-187-1	+	+	81	73	91	116	1549	3021	2948	5518	5404	3688	0.2	−1.2
IR90266-B-357-1	H	+	88	76	101	120	2543	3272	2422	5603	5786	3926	0.3	−3.0
IR55419-04			81	71	89	101	1825	2250	2824	4711	4076			
TDK1			99	81	73	97	173	2306	896	5985	5054			
Trial mean			86	74	97	109	1556	2547	2116	5237	4965			
SED			2	2	5	7	357	537	466	789	629			
P value			****	****	****	****	****	****	****	****	****			

LSS: lowland severe stress; LMS: lowland moderate stress; LNS: lowland non-stress; PC: principal component; +: with QTL; -: without QTL; H: heterozygote; P: probability of difference between genotypes; ****: significant at 0.01% *P* levels.

**Figure 4:**
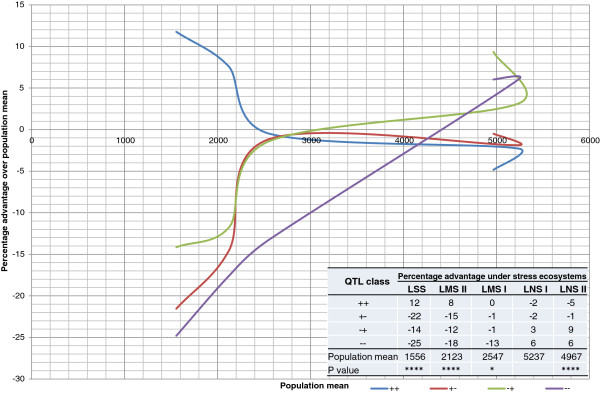
**QTL effect curve of lines with different combinations of *qDTY***_***3.1 ***_**and *qDTY***_***6.1 ***_**across lowland stress and non-stress conditions.** LSS: lowland severe stress; LMS: lowland moderate stress; LNS: lowland non-stress; ++: lines with *qDTY*_*3.1*_ and *qDTY*_*6.1*_; +−: lines with *qDTY*_*3.1*_ only; −+: lines with *qDTY*_*6.1*_ only; and −: lines without both *qDTY*_*3.1*_ and *qDTY*_*6.1*_. P: Probability of difference between genotypes; *, ****: significant at 5%, and 0.01% *P* levels, respectively.

To test the effect of these combinations on the whole population, a class mean analysis was conducted. Figure 4 shows the percentage yield advantage of four classes formed by the combination of *qDTY*_*3.1*_ and *qDTY*_*6.1*_ over population means across five lowland experiments. Mean yield from four QTL classes (based on peak marker) namely ++ (with *qDTY*_*3.1*_ and *qDTY*_*6.1*_), + − (with *qDTY*_*3.1*_ only), − + (with *qDTY*_*6.1*_ only) and -- (without both QTL) were used to calculate the percentage advantage of these classes over the population mean of varying stress and non-stress experiments conducted under lowland conditions. In agreement with the stability analysis, the lines possessing both QTL (++) showed the highest advantage under severe-stress conditions (12%) and performed stably across all experiments. The percentage advantage curve for the ++ lines stayed above or close to the X axis in all experiments. The effect of QTL in the ++ class gradually declined with decreasing severity of stress. Reduction in yield of up to 5% was also observed in this class under non-stress conditions. The classes + − and − + showed a higher degree of fluctuation in its percentage advantage curve, indicating lower stability compared with the ++ class. The lines with *qDTY*_*6.1*_ only showed decreased yield, compared with population means under severe- and moderate-stress conditions, while GY increased by up to 9% under non-stress conditions. This also showed that decline in yield under non-stress conditions in the lines with both QTL was due to *qDTY*_*3.1*_. In the lines that had only *qDTY*_*3.1*_, a decline in grain yield compared with population means was seen under both stress and non-stress conditions. However, both classes with single QTL (i.e., either *qDTY*_*3.1*_ or *qDTY*_*6.1*_) showed an advantage over lines without both QTL (−−) under all three stress conditions. The absence of *qDTY*_*3.1*_ in this class again led to the advantage under non-stress conditions, confirming the results from the previous classes.

### Effect of QTL across DTF classes

**Figure 5:**
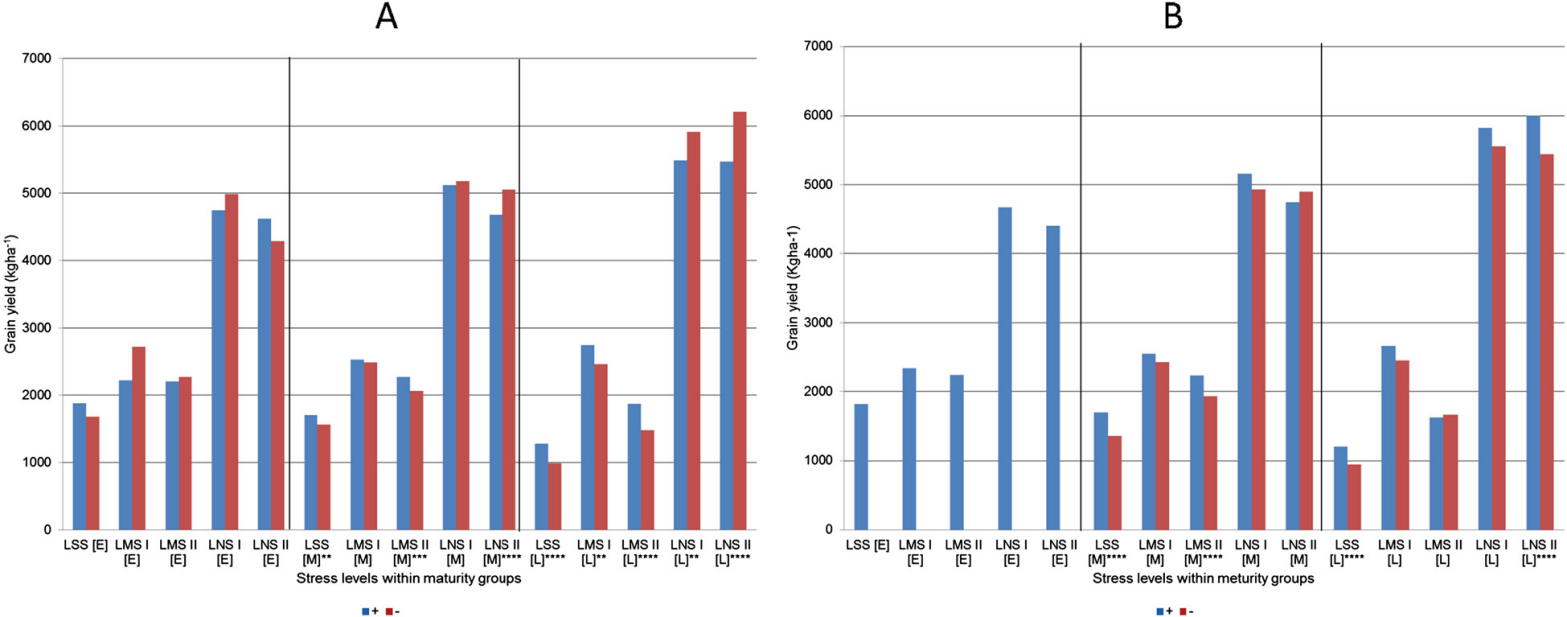
**Effect of *qDTY***_***3.1 ***_**(A) and *qDTY***_***6.1 ***_**(B) on grain yield of different maturity classes under lowland drought stress and non-stress conditions.** LSS: lowland severe stress; LMS: lowland moderate stress; LNS: lowland non-stress. E: early lines (Mean DTF ≤70 days under non-stress), M: Medium lines (Mean DTF = 71-75 days under non-stress), L: late lines (Mean DTF ≥76 days under non-stress). **, ***, ****: significant at 1%, 0.1%, and 0.01% *P* levels, respectively.

Due to the specific degree of the effects of *qDTY*_*3.1*_ and *qDTY*_*6.1*_ on grain yield under varying stress levels (Figure 4) and their effect on both DTF (Table 2), a test of QTL effect of both *qDTY*_*3.1*_ and *qDTY*_*6.1*_ was conducted across maturity groups to further refine these effects. The aim of the test was to understand the effect of the two QTL in lines exposed to different durations of stress. Lines were divided into three maturity classes: early (65–70 days), medium (71–75 days), and late (76–80 days) based on DTF under non-stress conditions (Figure 5). For *qDTY*_*3.1*_, the early lines with the QTL (+) showed higher yield under LSS conditions while early lines without the QTL (−) showed higher yield under LMS I, LMS II, and LNS II (Figure 5A). Clearer yield trends were observed under the medium and late lines, where the + lines showed higher yield under LSS, LMS I, and LMS II conditions while - lines showed higher yield under LNS I and LNS II conditions. For *qDTY*_*6.1*_, a reverse trend was observed in the medium and late lines, where the + lines yielded higher than the - lines in all stress conditions and in the non-stress condition (except for medium lines under LNS I). The - line class was not available in the early set (Figure 5B).

### QTL segment analysis of *qDTY*_*3.1*_

**Figure 6:**
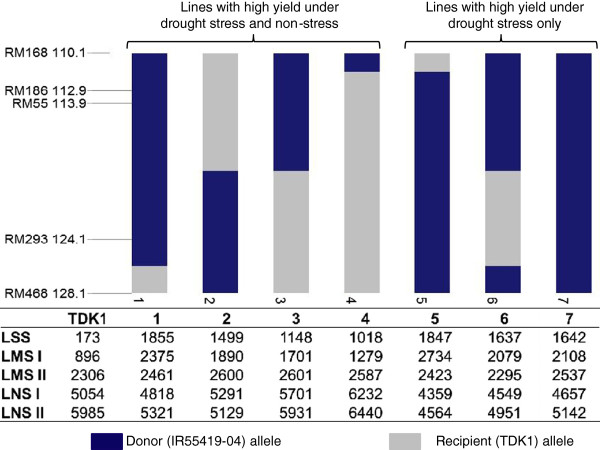
**Mean grain yield of lines with different segments of *qDTY***_***3.1 ***_**region under drought stress and non-stress conditions.** LSS: lowland severe stress; LMS: lowland moderate stress; LNS: lowland non-stress.

A negative effect of *qDTY*_*3.1*_ was seen on GY under non-stress conditions. The QTL, however, showed a consistent effect under drought-stress conditions. A class analysis of different segments of *qDTY*_*3.1*_ was conducted to determine the exact marker position that led to this effect. Lines with different segments of *qDTY*_*3.1*_ were grouped together and the mean GY of each class was calculated under drought-stress and non-stress conditions (Figure 6). Seven line classes were created with 7, 4, 11, 3, 4, 18, and 80 lines within them, respectively. The seventh class had the full segment of the QTL (Figure 5) because more lines belonged to this class compared with classes 1–6, with recombination events at one or more loci within the QTL region. The seven classes showed clear patterns of yield response under varying stress levels and non-stress conditions. While all QTL classes showed an advantage over TDK1 under all levels of stress, classes 1, 5, and 7 showed the highest yield under severe-stress conditions. All three classes had the IR55419-04 allele between RM186 and RM293. Classes with the IR55419-04 allele at RM293 (class 2) and RM55 (class 6) followed the first three classes. These classes had the TDK1 allele at RM55 and RM293, respectively. Under non-stress conditions, all three lowest-yielding classes (classes 5, 6, and 7) had the IR55419-04 allele at RM468 while three out of the four classes (classes 1, 3, and 7) had the TDK1 allele at this locus.

## Discussion

Our study identified three QTL: *qDTY*_*3.1*_, *qDTY*_*6.1*_, and *qDTY*_*6.2*_ (Figure 2). Two of these QTL—*qDTY*_*3.1*_ and *qDTY*_*6.1*_—showed a consistent effect across seasons under lowland drought-stress conditions, while *qDTY*_*6.1*_ and *qDTY*_*6.2*_ showed an effect under both upland and lowland conditions (Table 2). The use of a large backcross population developed from the cross of a high-yielding cultivar (TDK1, in this case) and a drought-tolerant donor (IR55419-04, in this case) was found to be highly suitable for generating precise GY data to conduct BSA. As seen in previous studies, BSA proved to be suitable for the identification of markers linked to loci affecting GY under drought, resulting in savings against the cost of genotyping efforts [10,17]. *qDTY*_*3.1*_ and *qDTY*_*6.1*_ have previously been reported to affect GY under severe lowland drought and favorable upland conditions, respectively [10,11]. *qDTY*_*3.1*_ showed an effect on GY in Swarna [10] and BR11 (IRRI, unpublished) backgrounds under severe lowland drought stress. *qDTY*_*6.1*_ had shown effect on GY under aerobic non-stress condition in Swarna and aerobic stress and non-stress conditions in IR72 backgrounds [11]. This QTL region has also been reported to affect other traits. For example, Wissuwa et al. [18] reported QTL for dry weight and phosphorus uptake at 10 and 13 cM, respectively. This region is also reported to have a major QTL, HD3A, related to heading date [19]. Dasgupta et al. [20] reported a gene (*Astol*) at 12.4 cM on chromosome 6. More recently, QTL conferring higher biomass and panicle number m^-2^ under upland reproductive-stage drought stress conditions were also reported as from this region [9]. *qDTY*_*6.2*_ was identified for the first time affecting GY under upland and lowland drought-stress conditions in this study. However, a QTL linked to PH at maturity has previously been reported to be at 86.0 cM, between RM541 and RM5371 [9], adjacent to *qDTY*_*6.2*_ (peak position 76.4 cM, between RM121 and RM541). To the best of our knowledge, this is the first case in which a QTL related to GY *per se* under upland and lowland environments has been seen in this region. While *qDTY*_*3.1*_ and *qDTY*_*6.1*_ have been reported previously in a population derived from Apo/2*Swarna [10,11], Apo (the tolerant donor in the Apo/2*Swarna population in the study conducted by Venuprasad et al. [10,11] and IR55419-04 (the tolerant donor in the IR55419-04/2*TDK1 population in our study) are sister lines derived from reciprocal crosses of the same parents: UPLRi5 and IR12979-24-1. It is highly probable that these regions hold the same source of tolerant alleles in the ancestry of the two tolerant parents.

An attempt was also made in this study to understand the specific effect of these QTL on the stability of performance of the lines in terms of GY under drought (Figure 3, Table 3). It was found that lines with both *qDTY*_*3.1*_ and *qDTY*_*6.1*_ showed an effect across different stress levels, and showed the most stable performance (Figure 4). These lines showed the highest advantage under severe-stress conditions. This advantage gradually declined with decreasing severity of stress. A similar observation has been reported by Bernier et al. [15] for *qDTY*_*12.1*_. Under less severe stress, more lines are able to produce grain in a population regardless of the presence of a QTL, which could be a reason for the reduced effect of the QTL in these conditions. Another reason for this reduction of effect under milder stress could be the possible effect of these QTL on drought-induced traits which may be leading to their higher effect under severe-stress conditions. *qDTY*_*3.1*_ had a negative effect on grain yield under non-stress conditions, an observation that has been reported previously [10]. While this QTL also showed an effect on DTF, a class analysis of lines by maturity groups revealed the patterns of effect that this QTL had on GY under varying levels of stress as well as in non-stress conditions. This division clearly showed the difference in effect of *qDTY*_*3.1*_ on lines of varying maturity periods (Figure 5). The QTL showed an effect under LSS and LMS conditions for medium- and late-duration lines, and showed an effect on early lines only under LSS. A clearer pattern of reduced yield under LNS conditions was seen on medium- and late-duration lines while, for early lines, this trend was seen during only one season. *qDTY*_*6.1*_ followed a clearer trend of effect on yield in the three maturity groups, showing an effect across all stress and non-stress conditions except in LMS I on the medium-duration group. The trends of effect of both QTL across maturity groups clearly indicate that specific patterns of effect of QTL were more pronounced in the late-duration group. Analysis also helped in understanding why the combination of *qDTY*_*3.1*_ and *qDTY*_*6.1*_ led to a more stable performance across varying levels of stress and non-stress conditions. On one hand, *qDTY*_*3.1*_ led to reduction in DTF and higher GY under LSS and LMS conditions, on the other hand it led to yield reduction under LNS condition. This reduction in GY under non-stress conditions was compensated for by *qDTY*_*6.1*_, which resulted in stable yield in all conditions. Although the presence of *qDTY*_*6.1*_ alone lends an advantage across all conditions, the presence of *qDTY*_*3.1*_ led to a further increase in the tolerance of lines under stress conditions. Because of the specific degree of the effects of *qDTY*_*3.1*_ and *qDTY*_*6.1*_ across varying levels of stress severity, it became important to clearly understand the effects of these QTL across the population. Although DTF can be affected by several other QTL in the background, it was important to understand the degree of the effect that these two QTL had within different maturity groups in the population. This could help in understanding better the effects of these QTL on lines subjected to different duration of stress. While, QTL analysis gave an idea of the cumulative effect of QTL across the population, this test helped determine which maturity group showed the highest advantage due to these QTL. The effect of both QTL was seen under severe-stress conditions in all three maturity classes. Although, the late-duration lines showed the most advantage under drought-stress conditions because of both QTL, it was also observed that the negative effect of *qDTY*_*3.1*_ on GY under non-stress conditions was more pronounced in these lines (Figure 5). It is important that these interactions of QTL be understood before undertaking large-scale marker-assisted breeding programs so that lines with certain combinations of QTL are delivered to specific target environments. For example, in this case, lines with both *qDTY*_*3.1*_ and *qDTY*_*6.1*_ can be deployed to environments where stress is severe and early maturity is desirable, and GY can come close to 5000 kg ha^-1^ under non-stress conditions. Lines with only *qDTY*_*6.1*_ can be aimed at environments where drought stress is comparatively less severe.

To further understand the specific effects of segments of *qDTY*_*3.1*_ affecting GY under varying stress severities and non-stress conditions, classes with different segments of this QTL were analyzed (Figure 6). Analysis showed the advantage of the IR55419-04 allele at the peak QTL region (between RM186 and RM293) under severe-stress conditions. These line classes also showed an advantage over TDK1 under moderate-stress conditions. Under non-stress conditions, the presence of the IR55419-04 allele at RM468 seemed to cause a negative effect on GY, as all three classes that yielded low under non-stress conditions had this allele at RM468. The negative effect of the marker loci had previously been seen in *DTY* QTL. For example, the loci RM262 within *qDTY*_*2.1*_ and RM24334 within *qDTY*_*9.1*_ showed negative effects on GY under severe- and moderate-stress conditions in cases where the donor alleles were present at these loci [21]. It becomes important to identify lines free from such regions or to eliminate them during MAS in order to achieve maximum advantage with these QTL. In this study, such line classes (classes 1 – 4 in Figure 6) were identified within *qDTY*_*3.1*_ with advantage under both stress and non-stress conditions. Lines with semi-dwarf PH and early flowering were also identified within these classes, for direct testing in the target environments or for use as donors for MAS programs.

## Conclusions

A QTL mapping study conducted on a BC_1_F_3:4_ population, developed from the cross IR55419-04/2*TDK1, showed the presence of three QTL—*qDTY*_*3.1*_ (RM168 to RM468), *qDTY*_*6.1*_ (RM586 to RM217), and *qDTY*_*6.2*_ (RM121 to RM541). *qDTY*_*3.1*_ and *qDTY*_*6.1*_ showed a consistent effect across seasons under lowland drought-stress conditions while *qDTY*_*6.1*_ and *qDTY*_*6.2*_ showed effect under both upland and lowland conditions. The test of QTL effect and stability analysis of the lines showed the combination of these QTL to be the most advantageous across a wide range of stress levels and across upland and lowland ecosystems. *qDTY*_*3.1*_ and *qDTY*_*6.1*_, in particular, showed highly complementary effects on GY across stress and non-stress ecosystems. With a clear understanding of the effects of these QTL made possible through this study, a large-scale QTL pyramiding program is now in its final stages where lines that have these QTL, with *Sub1* in the background of the recipient variety TDK1 are being developed and tested. This program aims for the development of drought- and submergence-tolerant versions of TDK1, which may lead to its wider adaptation and sustainable yields in case of flooding or drought events in the target environment.

## Methods

Our study presents results obtained from six experiments conducted at the Experiment Station of the International Rice Research Institute (IRRI), Los Baños, Laguna, Philippines, during the dry seasons (DS) and wet seasons (WS) of 2011,2012 and 2013. IRRI is located at 14°13′ N and 121°15′ E, at an elevation of 21 m above mean sea level. The soil type is Maahas clay loam, isohyperthermic mixed typic Tropudalf.

### Plant materials

A BC_1_F_3:4_ population developed from the cross IR55419-04/2*TDK1 was used in this study. The population, consisting of 365 lines, was evaluated in drought-stress and non-stress experiments conducted under lowland conditions in the DS2011, DS2012, and DS2013. A subset of 100 lines from this population was evaluated in upland (drought stress) conditions through experiments held in the WS2012. The drought-tolerant donor IR55419-04 in this population is an upland-adapted *indica* variety developed at IRRI, while the recipient TDK1 is a lowland-adapted high-yielding *indica* variety from Lao PDR.

### Experimental details

#### Upland and lowland conditions; stress (mild, moderate, and severe) and non-stress environments

The term upland in this study refers to field experiments conducted under direct-seeded, non-puddled, non-flooded, and aerobic conditions in levelled upland fields; lowland refers to field experiments conducted under flooded, puddled, transplanted, and anaerobic conditions. The term non-stress is used for experiments conducted under irrigated conditions with no drought stress, whereas experiments in which drought stress was imposed during the reproductive stage of the crop are referred to as stress experiments. Both definitions apply in both upland and lowland conditions. Stress is further classified into mild, moderate, and severe, based on the percentage of yield reduction compared with GY from non-stress experiments [6]. Under lowland conditions, stress experiments showing a yield reduction of 30% or less are termed mild stress (LMiS), those with a reduction of 31 – 65% are termed moderate stress (LMS), and those with yield reduction above 65% are referred to as severe stress (LSS) experiments. This study also presents the results from an upland stress experiment conducted in WS2012. In the absence of an upland non-stress counterpart of this experiment, the percentage of yield reduction compared with the mean yield of a lowland non-stress experiment was used to classify this experiment into upland mild-stress experiment (UMiS).

#### Phenotyping of mapping populations

This study presents the results from six experiments (three stress experiments and two non-stress experiments under lowland conditions, and one stress experiment under upland conditions). The experiments under lowland conditions were conducted with a set of 380 lines (365 BC_1_F_3:4_ lines + parents IR55419-04 and TDK1) in a 38 × 10 α lattice design with two replications (Table 1). Plot size for the lowland stress experiment was 1 m^2^ in the DS2011 and 2 m^2^ in the DS2012 and DS2013 stress and non-stress experiments. The upland experiment was conducted with 120 lines (100 BC_1_-derived lines + parents) in a 12 × 10 α lattice design with two replications (Table 1). The plot size of the upland experiment was 1 m^2^.

#### Management of upland and lowland experiments

For all lowland experiments, seeds were sown in a raised-bed nursery and 21-day-old seedlings were transplanted on the main field at one seedling per hill. After transplanting, approximately 5 cm of standing water was kept on the field until drainage before stress initiation at 30 days after transplanting (DAT) for stress experiments, while standing water was kept for up to 10 days before harvest in the non-stress experiments. Field management of lowland experiments was carried out as described by Venuprasad et al. [10].

Under upland conditions, seeds were dry-direct-seeded in aerobic soil at a seeding rate of 2.5 g per linear meter of row. The frequency of the stress cycle was not high in the WS experiment conducted using the IR55419-04/ 2*TDK1 population, and the experiment was irrigated only during prolonged dry spells when soil water tension fell below −50 kPa. This type of cyclic stress is reported to be efficient in screening for drought tolerance in populations consisting of genotypes with a broad range of growth duration [22], and ensures that all lines receive adequate stress during reproductive development. Field management of upland experiments was done as described by Bernier et al. [9].

### Data collection

In all lowland experiments, data on days to 50% flowering (DTF), plant height (PH) at maturity, and grain yield (GY) were recorded. DTF was recorded as the number of days from sowing up to the day on which 50% of the plants had flowering tillers. PH of three plants from each plot was measured at maturity from ground level to the tip of the tallest tiller and averaged to get the mean PH for analysis. GY from each plot was harvested at physiological maturity, dried to moisture content of 14%, and weighed [10]. This data set was then used to calculate for GY of the genotypes in kg ha^-1^ and then used for analysis.

### Statistical analysis

#### Mean and heritability calculations

Data from all experiments for computation of means and standard error of difference (SED) were analyzed using CROPSTAT version 7.2.3 (http://bbi.irri.org/products). Mixed model analysis of data was carried out using the model

$$y_{\text{ijk}}=\mu +g_{i}+r_{j}+b_{\text{lj}}+e_{\text{ijk}}$$

where *μ* is the overall mean, *g*_*i*_ is the effect of the i^th^ genotype, *r*_*j*_ is the effect of the j^th^ replicate, *b*_*lj*_ is the effect of the l^th^ block within the j^th^ replicate, and *e*_*ijk*_ is the error. Combined analysis was conducted on data from the lowland experiments to obtain line means across years under stress and non-stress conditions. Genotypic effects were considered fixed and replicates and block effects random. Broad-sense heritability (*H*) of the traits for single years was calculated as shown below

$$H=\frac{\sigma_{G}^{2}}{\sigma_{G}^{2}+\left[\frac{\sigma_{E}^{2}}{r}\right]}$$

where $\sigma_{G}^{2}$ is the genetic variance, $\sigma_{E}^{2}$ is the plot residual variance, and *r* is the number of replications. Variance components were calculated using the REML algorithm of PROC VERCOMP of SAS V.9.1 [23].

### GXE and stability analysis

GXE analysis was conducted using CROPSTAT version 7.2.3 using the model

$$y_{\text{ijkl}}=\mu +l_{j}+r_{\text{kj}}+b_{\text{lkj}}+g_{i}+{\left(\text{gl}\right)}_{\text{ij}}+e_{\text{ijkl}}$$

where *μ* is the overall mean, *l*_*j*_ is the effect of the j^th^ environment, *r*_*kj*_ is the effect of the k^th^ replicate within the j^th^ environment, *b*_*lkj*_ is the effect of the l^th^ block within the k^th^ replicate of the j^th^ environment, *g*_*i*_ is the effect of the i^th^ genotype, *(gl)*_*ij*_ is the effect of the interaction between the i^th^ genotype and the j^th^ environment, and *e*_*ijkl*_ is the error. The effects of genotype and interaction between genotype and environment were considered fixed while the other effects were considered random.

Stability of the genotypes across different environments was determined through the Additive Main effects and Multiplicative Interactions (AMMI) model [24,25] which can be written as

$$y_{\text{ij}}=\mu +g_{i}+e_{j}+\sum_{k=1}^{m}\ell_{k}u_{\text{ki}}^{*}v_{\text{kj}}^{*}+\epsilon_{\text{ij}}$$

where *y*_*ij*_ is the mean yield of i^th^ genotype in the j^th^ environment. *μ* is the general mean yield, *g*_*i*_ is i^th^ genotypic effect, *e*_*j*_ is the j^th^ location effect. ***ℓ***_k_ is the eigen value of the PCA axis k. $u_{\text{ki}}^{*}$ and $v_{\text{kj}}^{*}$ are the i^th^ genotype and j^th^ environment PCA scores for PCA axis k. ϵ_ij_ is the residual error and m is the number of PCA axes retained in the model.

### Genotyping of mapping populations

#### Generation of genotypic data

All DNA marker work was conducted at the Molecular Marker Applications Laboratory (MMAL) of the Plant Breeding, Genetics, and Biotechnology (PBGB) Division of IRRI. Fresh leaves from all lines were collected and freeze-dried. DNA was extracted from freeze-dried leaf samples using a modified CTAB method in deep-well plates. Polymerase chain reaction (PCR) was performed in 96-well polycarbonate plates using the method described by Panaud et al. [26]. Polyacrylamide gel electrophoresis [27] was then used for size separation of the amplified DNA fragments using a Mini-Vertical Electrophoresis System (CBS Scientific, model MGV-202-33). DNA fragments were then stained with SYBR Safe and visualized with a UV trans-illuminator.

#### BSA, whole population genotyping and QTL analysis

A total of 600 rice simple sequence repeat (SSR) markers were tested for polymorphism between the two parents, IR55419-04 and TDK1. All markers were taken from the published rice genome maps [28] and their physical position (Mb) on the Nipponbare genome (http://archive.gramene.org/markers/) was used for an approximate estimation of cM distances by multiplying by a factor of 3.92. One hundred sixty-five SSR markers showed polymorphism between the two parents and were used to conduct a BSA with the two parents and two DNA bulks derived from 15 (~4%; as outlined by [10]) high-yielding and 15 low-yielding lines based on GY data of the LSS trial conducted in the DS2011. Markers showing a clear difference in the form of banding patterns coinciding with those of the parents and clearly visible band intensity between high- and low-tail bulks were identified and used to genotype the full population of 365 lines. Additional markers were added to the region to obtain a clear confidence interval for the QTL. CIM was performed using the software Q Gene 4.3.10 [29]. The LOD thresholds obtained correspond to an experiment-wise type I error rate of 0.01 by running 1000 permutations. Graphical genotyping software GGT 2 [30] was used to construct a chromosome map of the line classes with different segments of the chromosome.

## Competing interests

The authors declare that they have no competing interests.

## Authors’ contributions

SD contributed to concept, design of experiment, data analysis, drafting and revision of the manuscript. AS contributed to data analysis and revision of the manuscript. MTSC, PTM and MA contributed in conducting the experiment in the field. AK conceived and designed the experiment, contributed to drafting and revision of the manuscript. All authors read and approved the final manuscript for publication.

## Supplementary Material

Additional file 1: Table S1 Grain yield data for lines used to develop the high- and low-yielding bulks across different seasons under drought-stress and non-stress conditions. LSS: lowland severe stress; LMS: lowland moderate stress; LNS: lowland non-stress; UMiS: upland mild stress; ^ǂ^: trials showing significant effect of one or more QTL. Click here for file
