# Supplementary material for: Multiple major QTL lead to stable yield performance of rice cultivars across varying drought intensities
Source: BMC Genet. 2014 Feb 3;15:16. doi: 10.1186/1471-2156-15-16 (PMC3933981; doi:10.1186/1471-2156-15-16)
Supplement: Additional file 1: Table S1 — Grain yield data for lines used to develop the high- and low-yielding bulks across different seasons under drought-stress and non-stress conditions. LSS: lowland severe stress; LMS: lowland moderate stress; LNS: lowland non-stress; UMiS: upland mild stress; ǂ: trials showing significant effect of one or more QTL. [file 1471-2156-15-16-S1.pdf]

Lines	LSS[‡]	LMS I	LMS II[‡]	LNS I	LNS II	UMiS[‡]	Bulk class
IR 90266-B-111-1	2572	2260	2254	5297	5580	3422	High yielding bulk
IR 90266-B-177-1	2462	.	.	5164	4511	4347	High yielding bulk
IR 90266-B-183-1	2556	2855	2689	4696	4795	6275	High yielding bulk
IR 90266-B-197-1	2552	2686	2162	6483	5152	4644	High yielding bulk
IR 90266-B-228-1	3027	2789	.	5165	5380	4682	High yielding bulk
IR 90266-B-243-1	2575	2764	1636	5754	6029	3522	High yielding bulk
IR 90266-B-343-1	2503	2624	2474	5037	4315	5381	High yielding bulk
IR 90266-B-357-1	2543	3272	2422	5603	5786	4559	High yielding bulk
IR 90266-B-390-1	2397	2693	1884	5678	6175	2406	High yielding bulk
IR 90266-B-398-1	2688	2325	2655	5347	5560	5399	High yielding bulk
IR 90266-B-446-1	2715	2136	2554	5409	4843	5842	High yielding bulk
IR 90266-B-450-1	2545	3033	3001	4629	4503	4945	High yielding bulk
IR 90266-B-484-1	2401	3130	1944	4616	5136	4519	High yielding bulk
IR 90266-B-492-1	2604	2160	1485	5392	5153	3181	High yielding bulk
IR 90266-B-519-1	2479	2577	2202	6337	4802	4483	High yielding bulk
**Mean (high yielding bulks)**	**2575**	**2665**	**2259**	**5374**	**5181**	**4507**	
IR 90266-B-106-1	483	2983	1350	.	6621	2136	Low yielding bulk
IR 90266-B-108-1	146	1441	1905	5275	5656	411	Low yielding bulk
IR 90266-B-123-1	409	2510	.	6903	6624	1912	Low yielding bulk
IR 90266-B-129-1	326	2787	.	6475	7140	3764	Low yielding bulk
IR 90266-B-134-1	194	2840	588	5462	5595	1042	Low yielding bulk
IR 90266-B-180-1	362	2304	1587	5262	5937	1102	Low yielding bulk
IR 90266-B-191-1	96	2484	2010	5636	5980	3377	Low yielding bulk
IR 90266-B-194-1	381	2857	1929	4767	6067	1577	Low yielding bulk
IR 90266-B-276-1	208	.	.	5398	6007	3263	Low yielding bulk
IR 90266-B-294-1	346	3064	605	.	6102	3134	Low yielding bulk
IR 90266-B-414-1	260	2879	1142	6254	5893	2837	Low yielding bulk
IR 90266-B-42-1	324	1814	1209	6573	5009	1663	Low yielding bulk
IR 90266-B-87-1	407	.	.	5989	6399	2718	Low yielding bulk
IR 90266-B-9-1	551	2299	.	7174	7164	3047	Low yielding bulk
IR 90266-B-93-1	379	3793	.	.	6173	3518	Low yielding bulk
**Mean (low yielding bulks)**	**325**	**2620**	**1370**	**5931**	**6158**	**2367**	
